# Targeting Host Metabolic and Epigenetic Rewiring Blocks Lytic Gammaherpesvirus Production

**DOI:** 10.3390/v18050574

**Published:** 2026-05-19

**Authors:** Morgan C. Jones, Tina M. Le, Connor J. Mahoney, Sara K. Hartman, Robynne D. Dona, Yennifer A. Gaspar, Sennah J. Hong, Benjamin R. Sheirbon, Thelma M. Escobar, Tracie Delgado

**Affiliations:** 1 Department of Biochemistry, University of Washington School of Medicine, Seattle, WA 98195, USA; 2 Institute for Stem Cell and Regenerative Medicine, University of Washington School of Medicine, Seattle, WA 98109, USA; 3 Department of Biology, Seattle Pacific University, Seattle, WA 98059, USA

**Keywords:** gammaherpesvirus, herpesvirus, murine herpesvirus 68, MHV-68, RNA-seq, metabolomics, chromatin, transcriptomics, antiviral, pentose phosphate pathway

## Abstract

Gammaherpesviruses are oncogenic viruses that reprogram host cell metabolism to support viral production. Among these, murine herpesvirus 68 (MHV-68) serves as a model system for studying lytic gammaherpesvirus infection and associated host cell changes. To characterize host transcriptional alterations induced throughout lytic gammaherpesvirus infection and identify novel host pathways that may be therapeutically targeted, we performed temporal bulk RNA-sequencing of mock- and MHV-68-infected NIH 3T3 cells at various timepoints throughout the lytic cycle. Our analysis revealed widespread and progressive host gene expression changes, including robust innate immune pathways and extensive remodeling of metabolic gene expression. We further identified a strong activation of the pentose phosphate pathway (PPP) genes, accompanied by increased abundance in PPP metabolic intermediates. Pharmacological inhibition of the PPP with 6-aminonicotinamide (6-AN) reduced infectious virus production. Moreover, at the intersection of metabolic and transcriptional reprogramming, we identified infection-associated gene expression changes in chromatin-modulating enzymes, including *Tet2*, and their metabolite co-factors, such as α-KG. Pharmacological inhibition of Ten-Eleven Translocation (TET) enzymatic activity led to a marked decrease in infectious MHV-68 production. Collectively, these findings define a novel metabolic–epigenetic crosstalk that supports productive gammaherpesvirus replication and identifies host pathways that can be targeted to treat lytic gammaherpesvirus infections.

## 1. Introduction

Approximately 10–15% of all human cancers are caused by viral infections [1,2,3]. Of the seven oncogenic viruses that are known to cause cancer in humans, two are members of the gammaherpesvirus family: Epstein–Barr Virus (EBV) and Kaposi’s Sarcoma-associated Herpesvirus (KSHV) [4]. EBV infects more than 90% of the global population and is estimated to contribute to 130,000–200,000 global cancer cases annually, including Hodgkin’s lymphoma, Burkitt’s lymphoma, and nasopharyngeal cancers [5]. KSHV infection causes Kaposi’s Sarcoma and remains one of the most common cancers in sub-Saharan Africa [6]. However, because humans are the only natural hosts for EBV and KSHV, this limits the ability to study human gammaherpesvirus pathogenesis and tumorigenesis in vivo [7].

Murine Herpesvirus 68 (MHV-68; murid herpesvirus 4), a gammaherpesvirus that is genetically and biologically related to KSHV and EBV [8], naturally infects rodents and establishes both acute and persistent infection in laboratory mice [9]. As such, MHV-68 serves as a valuable animal model for studying gammaherpesvirus pathogenesis and virus–host interactions relevant to human disease [10]. In contrast to EBV and KSHV, MHV-68 naturally and efficiently undergoes lytic replication in a variety of cell lines in vitro and readily forms plaques on cell monolayers [11,12]. As lytic replication is essential for viral dissemination and persistence, MHV-68 provides a robust and experimentally tractable system for investigating host cell changes throughout the lytic phase of gammaherpesvirus infections.

Viruses lack their own metabolism and therefore must hijack host cell metabolic machinery to generate ATP and biosynthetic precursors for their replication [13]. We previously demonstrated that both latent and lytic KSHV infections induce and require host cell metabolic reprogramming [14,15,16]. We found that latent KSHV infection induces glucose and lipid metabolism in host cells, and these metabolic alterations are required for latent-infected cell survival [14,15]. Additionally, we found glycolysis, glutaminolysis, and fatty acid synthesis are required for lytic KSHV replication and infectious virus production [16]. We also published the first lytic gammaherpesvirus metabolomics and lipidomics study, demonstrating that MHV-68 infection rewires host cell glucose, glutamine, lipid, and nucleotide metabolism [11]. However, how these metabolic changes are coordinated with host transcriptional reprogramming throughout the lytic gammaherpesvirus infectious cycle remains poorly defined.

Whole genome sequencing technologies are powerful tools for both identifying global changes in host gene expression during viral infection and aiding in the discovery of host pathways in response to infection [17,18,19]. To characterize host transcriptomic alterations induced upon MHV-68 infection, we performed bulk RNA-sequencing (RNA-seq) to generate a transcriptional map of host cell gene expression changes across the lytic MHV-68 infectious cycle. We identified several metabolic pathways that are altered, including the pentose phosphate pathway, whose pharmacological inhibition attenuated infectious MHV-68 production. We additionally identified key epigenetic mechanisms modulated during MHV-68 infection and demonstrate that inhibition of Ten-Eleven Translocation (TET) methylcytosine dioxygenase enzymes decreases infectious MHV-68 production. Collectively, this work provides a comprehensive resource defining host transcriptional and metabolic remodeling throughout lytic gammaherpesvirus infection and identifies host pathways that are potential antiviral targets to treat gammaherpesvirus infections.

## 2. Materials and Methods

### 2.1. Cell Lines, Viruses, and Reagents

Mouse fibroblast (NIH 3T3) cells (ATCC no. CRL-1658, Manassas, VA, USA) or African green-monkey kidney (Vero) cells (ATCC no. CCL-81, Manassas, VA, USA) were cultured at 37 °C and 5% CO_2_ in complete Dulbecco’s Modified Eagle Medium (DMEM) containing high glucose, L-glutamine, sodium pyruvate (Genesee no. 25-500, El Cajon, CA, USA), 1% penicillin streptomycin (Genesee no. 25-512, El Cajon, CA, USA), and 10% serum. NIH 3T3 cells were maintained in 10% newborn calf serum (Thermo Fisher Scientific no. 16010159, Pittsburgh, PA, USA) and Vero cells were maintained in 10% fetal bovine serum (Genesee no. 25-550, El Cajon, CA, USA). MHV-68 viral stocks (ATCC no. VR-1465, Manassas, VA, USA) were propagated in NIH 3T3 cells or Vero cells as previously described [11]. Stock solutions of 6-Aminonicotinamide (6-AN) (Thermo Fisher Scientific no. AAL0669203, Pittsburgh, PA, USA) and Bobcat339 hydrochloride (Selleckchem no. S6682, Houston, TX, USA) were dissolved in dimethyl sulfoxide (DMSO) and stored at −80 °C [20].

### 2.2. RNA Isolation, Library Preparation, and RNA-Sequencing

NIH 3T3 cells were seeded at a density of 2.2 million cells per 10 cm dish approximately 4 h prior to infection. NIH 3T3 cells were mock- or MHV-68-infected at a multiplicity of infection (MOI) of 5 for 2 h in 3.5 mL of serum-free DMEM media at 37 °C. After infection, the media was aspirated and replaced with 10 mL complete DMEM. At 4, 8, 12, and 24 h post-infection (hpi), matching pairs of mock- or MHV-68-infected cells were harvested. The cells were washed with PBS, trypsinized, resuspended in complete DMEM, and pelleted by centrifugation at 300× *g* for 5 min at 20 °C. Cell pellets were stored at −80 °C until processing. Samples were harvested at each time point in four independent experiments. One replicate was excluded following quality control due to poor clustering in principal component analysis, resulting in three biological replicates used for RNA-seq analysis [20].

Total RNA was isolated using the RNeasy Mini Kit (Qiagen no. 74104, Germantown, MD, USA) with Qiashredder homogenization (Qiagen no. 79654, Germantown, MD, USA) and on-column DNAse treatment (Qiagen no. 79254, Germantown, MD, USA) according to the manufacturer’s instructions. RNA concentration and purity were assessed at 260 nm and 280 nm using the NanoDrop One spectrophotometer (Thermo Fisher Scientific, Waltham, MA, USA). RNA integrity was evaluated using the Agilent High Sensitivity RNA ScreenTape assay (Agilent no. 5067-5579, Santa Clara, CA, USA) and the Agilent 4200 TapeStation. Approximately 500 ng of total RNA was used for library preparation using the Illumina Stranded mRNA Prep kit (Illumina No. 20040532, San Diego, CA, USA), which includes poly(A) selection. Libraries were indexed using the Illumina DNA/RNA UD Indexes Set A adapters (Illumina no. 20091646, San Diego, CA, USA). Library quality was assessed using the Agilent High Sensitivity D1000 ScreenTape Assay (Agilent no. 5067-5587, Santa Clara, CA, USA) and the Agilent 4200 TapeStation instrument. Library concentrations were determined using the Qubit dsDNA High Sensitivity Assay Kit (Thermo Fisher Scientific no. Q32854, Waltham, MA, USA) and Qubit 4 instrument according to the manufacturer’s instructions. Indexed libraries were pooled at equal molar concentrations and sequenced in the Illumina NextSeq 2000 using a P3 flow cell with up to 1.2B single reads (Illumina no. 20100989, San Diego, CA, USA) [20].

### 2.3. RNA-Sequencing Data Analysis

Raw FASTQ files were downloaded from Illumina BaseSpace. Sequencing quality was assessed using FastQC, version 0.12.0 (https://www.bioinformatics.babraham.ac.uk/projects/fastqc/; accessed 15 June 2025). Reads were aligned to the mouse reference genome using the splice-aware aligner STAR [21]. STAR genome indices were generated using the NCBI RefSeq-annotated mm39 genome assembly. Transcript quantification was performed using Salmon [22]. Following quantification, gene-level count data were imported into R for downstream analysis. Principal component analysis (PCA) was performed using deepTools2 [23] to assess sample clustering in paired mock- and MHV-68 infected samples while accounting for batch effects. One biological replicate did not cluster consistently with the remaining replicates and was excluded from further downstream analyses following quality control. Differential gene expression (DEG) analysis was performed using DESeq2 [24] on paired mock- and MHV-68-infected samples. A log 2 fold-change (log2FC) shrinkage was applied using the lfcShrink function with local fit parameters to reduce inflation of log2FC for low abundance transcripts. Raw *p*-values were calculated using the Wald test, and adjusted *p*-values (padj) were calculated using the Benjamini–Hochberg method to control the false discovery rate [25]. Genes with padj < 0.05 were considered differentially expressed.

### 2.4. RNA Seq Data Tables and Visualizations

The final analyzed RNA-seq data was exported as a CSV file and converted into a Microsoft excel worksheet (Supplementary Table S1). At all time points (4, 8, 12, and 24 hpi), mouse gene name, base mean (averaged across all time points), log2FC, and padj are included. The complete RNA-seq raw and analyzed data has been deposited in the NCBI Gene Expression Omnibus (GEO) under the accession number GSE314414. A metabolism gene-specific RNA-seq dataset (Supplementary Table S2) was also generated using the same methodology, with additional filtering to only include genes annotated in the KEGG metabolic pathway mmu01100 [26]. Furthermore, we filtered the RNA-seq dataset to only enzymes that mediate epigenetic modifications using a previously published dataset (Supplementary Table S3) [27]. A Venn diagram depicting overlaps of differentially expressed genes (padj < 0.05) at 4, 8, 12, and 24 hpi was generated using the InteractiVenn web application [28]. A bar graph quantifying the number of differentially expressed genes (upregulated vs. downregulated) was generated using Graphpad Prism 8 software. Volcano plots were generated using R, plotting log2FC vs. −log10(padj), with significance thresholds set to padj ≤ 0.05 and the specified log2FC cutoffs. RNA-seq heat maps depicting log2FC were generated using Graphpad Prism 8 software, with genes exhibiting base mean expression < 10 excluded.

### 2.5. Gene Ontology and Pathway Enrichment Analysis

Gene ontology and pathway enrichment analysis were performed using ShinyGO version 0.85 [29]. DEGs were filtered prior to enrichment analysis (padj ≤ 0.05) at indicated log2FC thresholds. Enrichment analyses were performed independently at each time point using the *Mus musculus* gene set (mmusculus_gene_ensembl; genome assembly GRCm39; taxonomy ID 10090; Ensembl). Pathway enrichment was restricted to the KEGG database. A false discovery rate (FDR) cutoff of 0.05 was applied. For visualization, lollipop plots displaying the top 20 enriched pathways (when available) were generated and sorted by fold enrichment along the x-axis. Dot color indicates −log10(FDR).

### 2.6. Metabolomics

Metabolomics data were obtained from our previously published lytic MHV-68 metabolomics dataset [11] and thus no new metabolomics experiments were conducted. For this study, we performed targeted re-analysis of our metabolomics dataset at 4, 8, 12, and 24 hpi post lytic MHV-68 infection of NIH 3T3 cells (MOI of 3). Metabolites were selected based on their involvement in the highlighted metabolic and epigenetic pathways revealed by our RNA-seq analysis, including the pentose phosphate pathway intermediates and metabolites associated with chromatin-modifying enzymes. Heat maps were generated to visualize log2FC in MHV-68-infected cells compared to mock-infected cells at 4, 8, 12, and 24 hpi. All metabolite normalization and statistical analysis were performed in the original study [11].

### 2.7. Drug Assays, Viral Titers, and Cell Viability

NIH 3T3 cells were seeded at a density of 760,000 cells in 6 cm dishes approximately 4 h prior to infection. NIH 3T3 cells were mock- or MHV-68-infected (MOI of 0.1) for 2 h in serum-free DMEM. A MOI of 0.1 was chosen to allow approximately two MHV-68 replication cycles in the presence and absence of a pharmacological inhibitor. Following infection, the media were aspirated and replaced with complete DMEM supplemented with either vehicle (DMSO control), 35 µM 6-AN, or 100 µM Bobcat339 for the duration of the experiment. At 48 hpi, cellular supernatants were cleared by centrifugation at 10,000 rpm and the resulting viral supernatant (extracellular virions) was frozen at −80 °C for future plaque assay analysis in Vero cells as previously described [11]. Plaque-forming units per mL (PFUs/mL) were calculated from duplicate wells using the equation: (dilution factor) × (1/0.2 mL) × (average pfu) = pfu/mL [20]. Post clearing of cellular supernatants by centrifugation, the remaining cell pellets were pooled with their matching trypsinized cell samples and centrifuged at 125× *g*. The final cellular pellets were resuspended in 600 µL complete DMEM. Cell number and viability were assessed using a trypan blue exclusion assay and the Biorad TC-20 automated cell counter (Bio-Rad no. 1450102, Hercules, CA, USA). Total cells, live cells, and percent viability were calculated by the Biorad TC-20 automated cell counter [20]. Viral titer bar graphs were generated using Graphpad Prism 8 software.

### 2.8. Nuclear Extraction and TET2 Western Blot

Mock- and MHV-68-infected NIH 3T3 cells (MOI  =  5) were trypsinized and pelleted by centrifugation (125× *g* for 5 min at 4 °C) at 24 hpi. Cell pellets were washed in phosphate-buffered saline and then pelleted by centrifugation (125× *g* for 5 min at 4 °C) before beginning nuclear extraction [30]. Cells were resuspended in hypotonic lysis buffer supplemented with protease inhibitors (10 mM TRIS pH 7.9, 1.5 mM MgCl_2_, 10 mM KCl, 0.5 mM DTT, 0.2 mM PMSF, 1 μg/mL Aprotinin, 1 μg/mL Leupeptin, 1 μg/mL Pepstatin, 0.4 mM Na_3_VO_4_, 0.2 mM Na_4_P_2_O_7_), incubated on ice for 10 min, and pelleted by centrifugation (500× *g* for 5 min at 4 °C). The supernatant was removed, the cells were resuspended in lysis buffer, transferred to a Wheaton Dounce Tissue Grinder (1 mL, loose pestle), and then fractionated with 10 strokes. The homogenate was centrifuged (500× *g* for 10 min at 4 °C) and the supernatant (cytosolic extract) and pellet (nuclei) were separated. The nuclei were resuspended in high-salt buffer with protease inhibitors (20 mM TRIS pH 7.9, 25% Glycerol, 1.5 mM MgCl_2_, 420 mM NaCl, 0.2 mM EDTA, 0.5 mM DTT, 0.5 mM PMSF, 1 μg/mL Aprotinin, 1 μg/mL Leupeptin, 1 μg/mL Pepstatin, 0.4 mM Na_3_VO_4_, 0.2 mM Na_4_P_2_O_7_) and solubilized by sonication using the Bioruptor Plus sonication device (Diagenode, Seraing, Belgium) for 6 cycles on high, 5 s on/15 s off. Protein was quantified using Pierce Bradford Plus Protein Assay Reagent (Thermo Fisher Scientific no. 23238, Waltham, MA, USA) according to the manufacturer’s specifications.

Solubilized nuclear extracts (25 μg) were separated by a sodium dodecyl sulphate polyacrylamide (SDS-PAGE) gel, transferred to a polyvinylidene difluoride membrane (2 h wet transfer at 100V), blocked with bovine serum albumin, and blotted with the appropriate primary antibody [1:1000 rabbit anti-TET2 (Cell Signaling Technology no. 36449, Danvers, MA, USA) and 1:2000 rabbit anti-H3 (Abcam no. 1791, Waltham, MA, USA)] and subsequently with horseradish peroxidase (HRP)-conjugated 1:10,000 goat anti-rabbit (Invitrogen no. G21234, Carlsbad, CA, USA). HRP-conjugated proteins were incubated in SuperSignal West Femto Maximum Sensitivity Substrate (Thermo Fisher Scientific no. 34094, Waltham, MA, USA) and Pierce ECL Western Blotting Substrate (Thermo Fisher Scientific no. 32106, Waltham, MA, USA). Immunoreactive proteins were detected using a ChemiDoc imager (Bio-Rad, no. 12003153, Hercules, CA, USA) with Image Lab Touch Software version 2.2.0.08.

### 2.9. Global TET Activity Assay

Mock- and MHV-68-infected NIH 3T3 cells (MOI  =  5) were trypsinized and pelleted by centrifugation (125× *g* for 5 min at 20 °C) at 24 hpi. Cell pellets were washed in phosphate-buffered saline and then pelleted by centrifugation (125× *g* for 5 min at 20 °C) before freezing at −80 °C for future processing. Genomic DNA isolation was performed using the Qiagen DNeasy Blood & Tissue Kit (Qiagen no. 69506, Germantown, MD, USA). Global TET activity measuring DNA demethylation of 5-methylcytosine (5-mC) to 5-hydroxymethylcytosine (5-hmC) was measured by Enzyme-Linked Immunosorbent Assay (ELISA) using the Global 5-hmC DNA ELISA Kit (Active Motif no. 55025, Carlsbad, CA, USA), with 300 ng of MesI digested genomic DNA added per well.

### 2.10. Statistical Analysis

Statistical analysis of viral titer bar graphs and Global TET activity were performed using Graphpad Prism and *p*-values were determined using paired Student’s (two-tailed) *t*-test. Standard errors of the mean (SEM) are shown. A *p*-value of <0.05 was considered statistically significant. RNA-seq and pathway enrichment statistics were performed as described above.

## 3. Results

### 3.1. Lytic Gammaherpesvirus Replication Reprograms Host Cell Gene Expression

**Figure 1:**
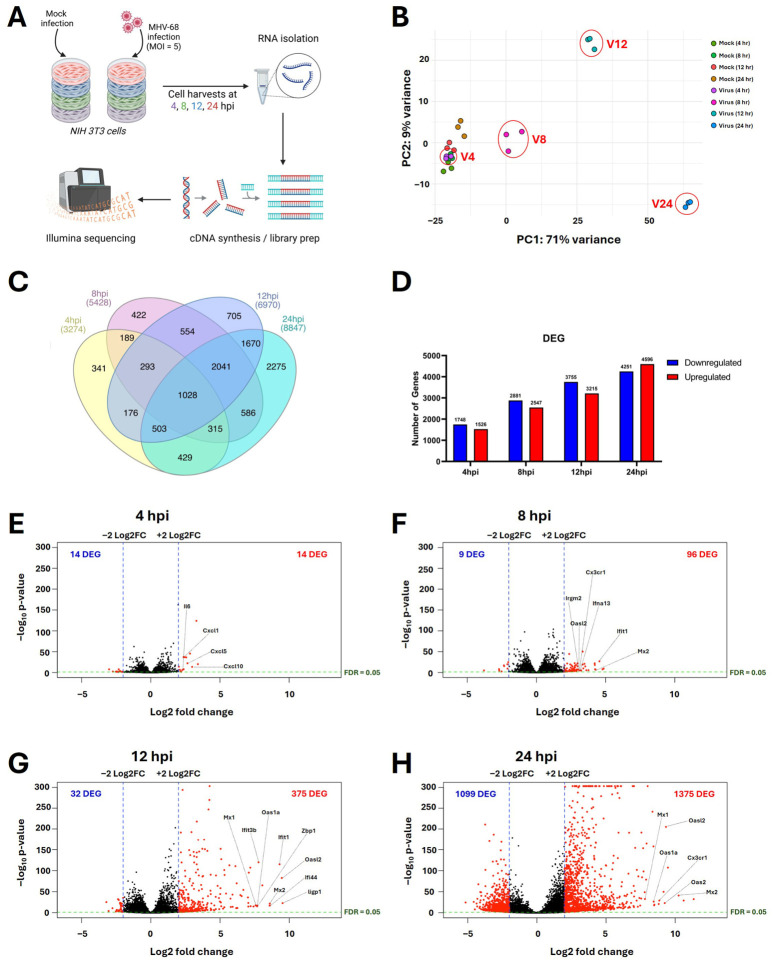
Lytic Gammaherpesvirus Infection Reprograms Host Cell Gene Expression. (**A**) Mock- or MHV-68-infected NIH 3T3 cells (MOI = 5) were harvested at 4, 8, 12, or 24 hpi in three biological replicates for RNA-seq analysis. Total RNA was isolated, followed by cDNA synthesis and library preparation. Sequencing was performed using the Illumina NextSeq 2000. Image created in Biorender. (**B**) PCA of mock- vs. MHV-68-infected cells (Virus [V]) at 4, 8, 12, and 24 hpi. (**C**) Venn diagram of DEGs in MHV-68-infected NIH 3T3 cells compared to mock-infected NIH 3T3 cells at 4, 8, 12, and 24 hpi (padj < 0.05). (**D**) Bar graphs depicting the number of downregulated (blue) vs. upregulated (red) DEGs in MHV-68-infected cells compared to mock-infected cells at 4, 8, 12, and 24 hpi (padj < 0.05). (**E**–**H**) Volcano plot analysis of DEGs at (**E**) 4 hpi, (**F**) 8 hpi, (**G**) 12 hpi, and (**H**) 24 hpi. DEGs with thresholds of log2FC > +/−2 and padj < 0.05 are highlighted in red. Top immune response genes are labeled.

To profile host cell transcriptional changes during lytic MHV-68 infection, we utilized NIH 3T3 mouse fibroblasts, a well-established cell line that supports robust MHV-68 replication to high titers [31,32]. Importantly, low-passage NIH 3T3 cells are non-transformed and exhibit contact inhibition [33,34], permitting assessment of virus-induced host transcriptional and metabolic remodeling in the absence of extensive oncogenic reprogramming. In vitro, the MHV-68 lytic cycle spans approximately 24–36 h, with temporally ordered viral gene expression: immediate-early (4 hpi), early (8 hpi), late (12 hpi), very late (24 hpi) [35,36,37]. Therefore, to capture host cell gene expression changes throughout infection, we conducted bulk RNA-seq in mock- vs. MHV-68-infected NIH 3T3 cell samples (MOI = 5) throughout the lytic MHV-68 cycle (4, 8, 12, and 24 hpi) (Figure 1A). Principal component analysis of three independent experiments revealed tight clustering of replicates (mock- vs. MHV-68-infected) at each time point (Figure 1B). Sample analysis using the DESeq2 R package revealed extensive host transcriptional reprogramming during gammaherpesvirus lytic infection (Supplementary Table S1). The overall number of DEGs (<0.05 padj) increased over time from 3274 (4 hpi) to 8847 (24 hpi) (Figure 1C,D, Supplementary Table S1). A core set of 1028 host genes were differentially expressed at all time points (Figure 1C).

Volcano plot analysis (≥2 log2FC) also demonstrated time-dependent increases in both the number and magnitude of transcriptional host changes as lytic replication progressed (Figure 1E–H), from 14 downregulated and 14 upregulated DEGs at 4 hpi (Figure 1E) to 1099 downregulated and 1375 upregulated DEGs by 24 hpi (Figure 1H). Examination of the top upregulated genes at each time point revealed a strong and temporally coordinated activation of innate immune gene transcripts (Figure 1E–H, Supplementary Table S1). During the early stage of gammaherpesvirus lytic replication (4 hpi) (Figure 1E), pro-inflammatory cytokine and chemokine transcripts *(Il6*, *Cxcl2*, *Cxcl5*, *Cxcl10*) were among the highest induced genes. By 8 hpi (Figure 1F), the transcripts of additional chemokine-related genes (*Cx3cr1*) and a type I interferon (*Ifna13*), along with several canonical interferon-stimulated genes (ISGs) (*Irgm2*, *Oasl2*, *Ifit1*, *Mx2*), emerged. From 12 to 24 hpi (Figure 1G,H), antiviral ISGs (*Ifit1*, *Ifit3b*, *Mx1*, *Mx2*, *Oas1a*, *Oasl2*, *Zbp1*, *Ifi44*, *ligp1*) dominated, demonstrating a sustained antiviral state during late-stage lytic gammaherpesvirus replication.

### 3.2. Lytic Gammaherpesvirus Infection Reprograms Host Signaling, Metabolic, and Transcriptional Pathways

**Figure 2:**
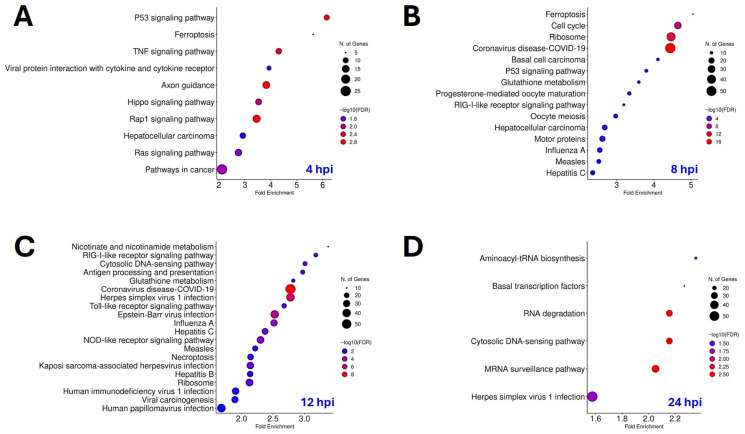
Host Transcriptional Pathways Activated During Gammaherpesvirus Lytic Infection. Gene ontology KEGG pathway enrichment analysis of upregulated DEGs in MHV-68-infected NIH 3T3 cells vs. mock-infected cells (log2FC ≥ +0.5; padj < 0.05) at (**A**) 4 hpi, (**B**) 8 hpi, (**C**) 12 hpi, and (**D**) 24 hpi using ShinyGO version 0.85. Dot size represents the number of mapped genes and dot color indicates −log10(FDR).

Gene ontology and KEGG enrichment analysis of upregulated genes at each time point identified the biological functions and pathways overrepresented at distinct stages of lytic gammaherpesvirus infection (Figure 2A–D). At 4 hpi (Figure 2A), early host responses during infection included enrichment of p53 signaling, ferroptosis, TNF signaling, and cytokine receptor interaction pathways. At 8 hpi (Figure 2B), the enrichment included the RIG-I-like receptor signaling immune pathway, p53 signaling pathway, glutathione metabolism, ferroptosis, and pathways linked to cell cycle and ribosome biogenesis. By 12 hpi (Figure 2C), a broader enrichment of immune response pathways emerged (RIG-I-like receptor, cytosolic DNA-sensing pathway, antigen processing and presentation, Toll-like receptor signaling pathway, NOD-like receptor signaling pathway). Notably, multiple viral infection KEGG modules were observed, including human gammaherpesviruses KSHV and EBV, revealing a conservation of host cell changes shared across gammaherpesvirus infections. By 24 hpi (Figure 2D), pathway enrichment shifted from immune activation to the remodeling of host transcriptional and RNA-processing machinery. Analysis of downregulated DEGs (Supplementary Figure S1) revealed multiple pathways linked to cytoskeletal organization and focal adhesion were downregulated in early infection (4 hpi). By 12 hpi, repression of cell cycle and DNA repair pathways were decreased. By 24 hpi, there was broader downregulation of protein processing, cytoskeletal organization, and focal adhesion, alluding to host transcriptional shutdown and cytoskeleton collapse.

### 3.3. Gammaherpesvirus Lytic Replication Modulates Host Cell Metabolic Pathways

**Figure 3:**
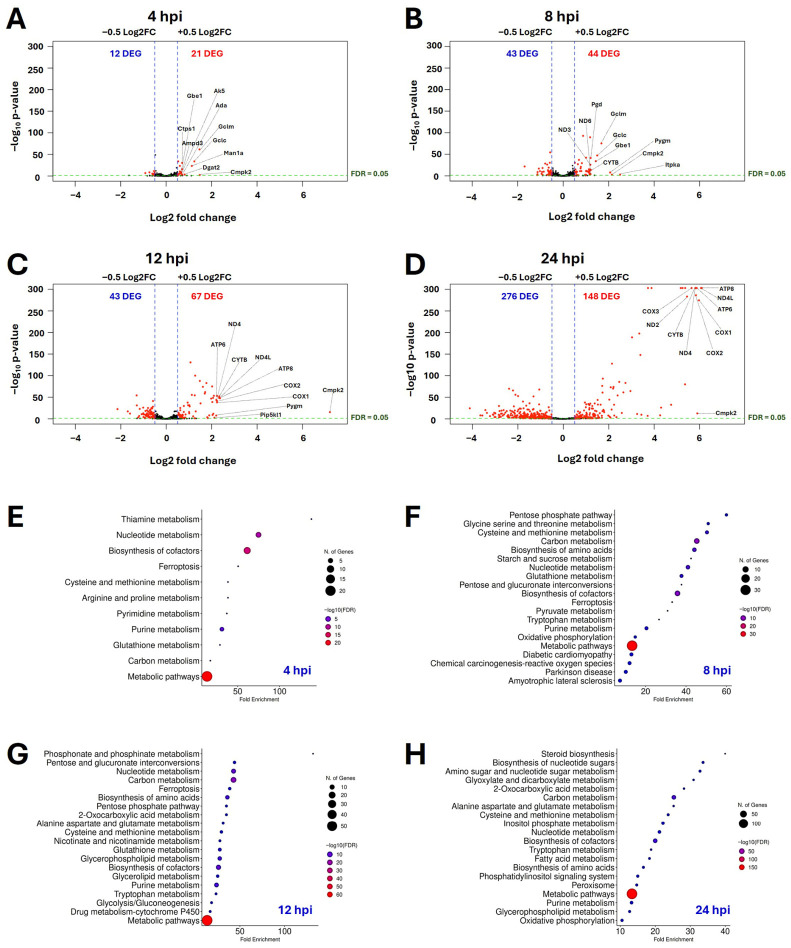
Lytic Gammaherpesvirus Infection Rewires Host Metabolic Gene Expression. (**A**–**D**) Volcano plots of metabolic DEGs (KEGG module mmu01100) in MHV-68-infected NIH 3T3 cells compared to mock-infected cells at (**A**) 4 hpi, (**B**) 8 hpi, (**C**) 12 hpi, and (**D**) 24 hpi. Genes with thresholds of log2FC > +/−0.5 and padj < 0.05 are highlighted in red. The top ten upregulated metabolic genes at each time point are labeled. (E-H) Gene ontology KEGG pathway enrichment analysis of upregulated metabolic DEGs (log2FC ≥ +0.5; padj < 0.05) at (**E**) 4 hpi, (**F**) 8 hpi, (**G**) 12 hpi, and (**H**) 24 hpi using ShinyGO version 0.85. Dot size represents the number of mapped genes and dot color indicates −log10(FDR).

Consistent with widespread transcriptional remodeling, gene ontology pathway analysis also revealed the enrichment of multiple metabolic pathways throughout lytic MHV-68 infection (Figure 2). To specifically assess metabolic reprogramming, we filtered the RNA-seq dataset to metabolic genes only using the global metabolic pathway KEGG mmu01100 dataset and then analyzed metabolic DEGs across all time points (Supplementary Table S2). Volcano plot analysis of metabolic transcripts showed a progressive and time-dependent increase in the number of metabolic DEGs (Figure 3A–D). Examination of the top ten upregulated metabolic genes at each time point revealed early nucleotide and redox metabolism reprogramming, which later shifted to mitochondrial electron transport chain reprogramming. For example, at 4 hpi (Figure 3A), the top upregulated metabolic transcripts during MHV-68 infection included nucleotide metabolism transcripts (*Ak5*, *Ada*, *Ctps1*, *Ampd3*, *Cmpk2*) and glutathione metabolism transcripts (*Gclm*, *Gclc*). By 24 hpi, mitochondrial encoded electron transport chain transcripts peaked (*ND2*, *ND4*, *ND4L*, *ATP6*, *ATP8*, *COX1*, *COX2*, *COX3*, *CYTB*).

To identify the top metabolic pathways, we performed KEGG enrichment analysis on upregulated metabolic DEGs at each time point (Figure 3E–H). While volcano plots highlight the most significantly altered individual genes, gene ontology KEGG enrichment analysis identifies biological pathways that are coordinately overrepresented among differentially expressed genes. At 4 hpi (Figure 3E), KEGG enrichment demonstrated increased thiamine, nucleotide, ferroptosis, amino acid, and glutathione metabolism pathways. At 8 hpi (Figure 3F), pentose phosphate pathway (PPP), amino acid, carbon, nucleotide, starch, sucrose, glutathione, ferroptosis, pyruvate, and oxidative phosphorylation metabolism pathways were increased. By 12 hpi (Figure 3G), nucleotide, amino acid, ferroptosis, carbon, glycolysis/gluconeogenesis, glutathione, 2-oxocarboxylic acid, nicotinate and nicotinamide, glycerophospholipid and glycerolipid metabolic pathways increased. By 24 hpi (Figure 3H), nucleotide metabolism, amino acid, fatty acid and steroid biosynthesis, carbon, 2-oxocarboxylic acid, glycophospholipid, inositol phosphate, and oxidative phosphorylation pathway transcripts increased. Analysis of downregulated metabolic DEGs (Supplementary Figure S2) demonstrated that early lytic infection (4 and 8 hpi) downregulated multiple metabolic pathways, including fatty acid biosynthesis, glycosaminoglycan biosynthesis, and amino acid metabolism. By 12 hpi, multiple glycan biosynthesis pathways were suppressed. During late lytic infection (24 hpi), metabolic repression expanded to include the TCA cycle, oxidative phosphorylation, and glycolysis. Taken together, these data revealed temporal patterns in host metabolic rewiring during lytic gammaherpesvirus infection.

### 3.4. Gammaherpesvirus Infection Activates and Requires the Pentose Phosphate Pathway for Infectious Virus Production

**Figure 4:**
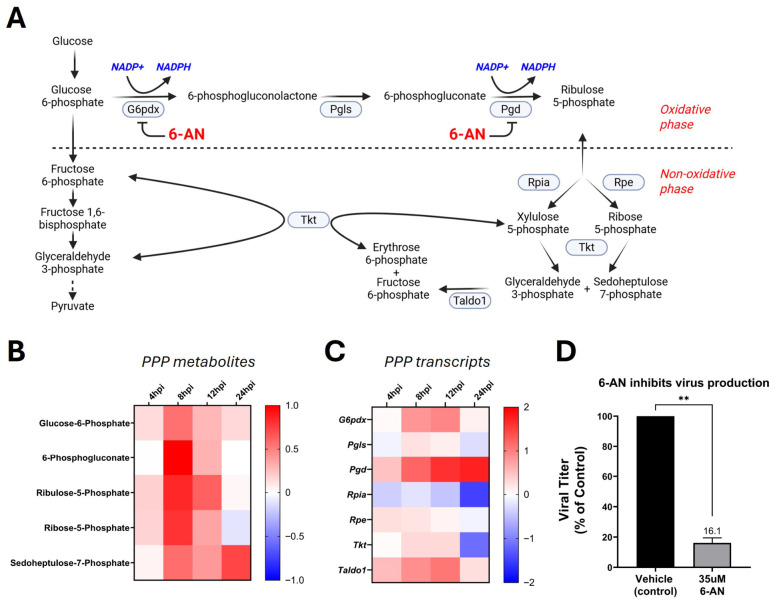
Gammaherpesvirus lytic infection Activates the Pentose Phosphate Pathway to Support Virus Production. (**A**) Schematic of the pentose phosphate pathway (PPP). In the oxidative phase, glucose-6-phosphate branches from glycolysis and is converted to 6-phosphogluconolactone by the enzyme glucose-6-phosphate dehydrogenase (*G6pdx*), followed by conversion to 6-phosphogluconate by the enzyme 6-phosphogluconolactonase (*Pgls*), and then to ribulose 5-phosphate by the enzyme 6-phosphogluconate dehydrogenase *(Pgd*). 6-Aminonicotinamide (6-AN), highlighted in red, is an inhibitor of the NADP+-dependent enzymes *G6pdx* and *Pgd*. NADPH-generating reactions are highlighted in blue. During the non-oxidative phase, ribulose 5-phosphate is interconverted into a variety of sugar phosphates, including ribose-5-phosphate for nucleotide synthesis and glycolytic intermediates (fructose-6-phosphate and glyceraldehyde-3-phosphate) via the transketolase (*Tkt*) and transaldolase (*Taldo1*) reactions. Metabolites are in black and enzymes are circled. Image created in Biorender. (**B**) Metabolomics analysis of PPP intermediates at 4, 8, 12, and 24 hpi in three biological replicates. The log2FC is shown, where red depicts an increase and blue shows a decrease in MHV-68-infected (MOI = 3) NIH 3T3 cells compared to mock-infected cells. (**C**) RNA-seq analysis of PPP transcripts at 4, 8, 12, and 24 hpi in three biological replicates. The log2FC is shown, where red depicts an increase and blue shows a decrease in MHV-68-infected (MOI = 5) NIH 3T3 cells compared to mock-infected cells. (**D**) NIH 3T3 cells were mock- or MHV-68-infected (MOI = 0.1) and treated with 35 µM 6-AN or vehicle (control) for 48 hpi in three biological replicates. Viral titers were quantified by plaque assays and normalized to vehicle controls. Paired Student’s *t*-test (Graphpad Prism); **, *p* < 0.01.

Due to the strong enrichment of various nucleotide biosynthetic pathways, we next performed a more detailed analysis of the PPP, a major source of ribose sugars needed for nucleotide synthesis. The PPP (Figure 4A) directly branches from glycolysis and contains both oxidative and non-oxidative phases. The oxidative phase generates NADPH (needed for glutathione antioxidant defense and lipid synthesis) and ribulose-5-phosphate (needed to build nucleotides). The non-oxidative phase converts excess ribose-5-phosphate back into glycolytic intermediates. Our metabolomics analysis revealed that key metabolites in the oxidative phase of the PPP (glucose-6-phosphate, 6-phosphogluconate, and ribulose-5-phosphate) were consistently elevated during MHV-68 infection, with peaks at 8–12 hpi (Figure 4B). Similarly, non-oxidative metabolites (ribose-5-phosphate and sedoheptulose-7-phosphate) increased during infection. Our transcriptomic analysis paralleled these metabolic trends. We observed an increase in oxidative PPP transcripts *G6pdx* (8 and 12 hpi), *Pgls* (8 hpi), and *Pgd* (4–24 hpi) during early and late lytic infection (Figure 4C). Non-oxidative PPP enzyme transcripts *Rpe* (4 and 8 hpi), *Tkt* (8 and 12 hpi), and *Taldo1* (4–24 hpi), were also induced during infection. Overall, these data demonstrate a global increase in the PPP which supplies ribose sugars, NADPH-reducing power, and glycolytic intermediates that can be used to support viral replication.

To test if the PPP is required for infectious viral production, NIH 3T3 cells were mock- or MHV-68-infected (MOI = 0.1) and then treated with vehicle (control) or 35 µM 6-aminonicotinamide (6-AN). The pharmacological inhibitor 6-AN blocks NADP+-dependent enzymes, *G6pdx* (rate-limiting enzyme) and *Pgd*, and therefore inhibits the oxidative branch of the PPP (Figure 4A) [38,39]. Treatment of NIH 3T3 cells with 6-AN had minimal cytotoxicity, with a 2% increase in cell death in mock-infected cells and a 3% increase in cell death in MHV-68-infected cells compared to vehicle-treated cells (solvent control) (Table 1). Overall, 35 µM 6-AN treatment of MHV-68-infected cells resulted in a ~84% reduction in infectious virus production compared to vehicle-treated cells (*p* < 0.01) (Figure 4D). Due to inhibition of cell proliferation by 35 µM 6-AN treatment, normalization of viral titer to live cell number revealed 35 µM 6-AN treatment blocks infectious virus production by ~5-fold compared to vehicle-treated cells (Table 1). Taken together, these data suggest the induction of the PPP by MHV-68 lytic infection is required for virion production.

### 3.5. Gammaherpesvirus Infection Modulates Epigenetic Mechanisms

**Figure 5:**
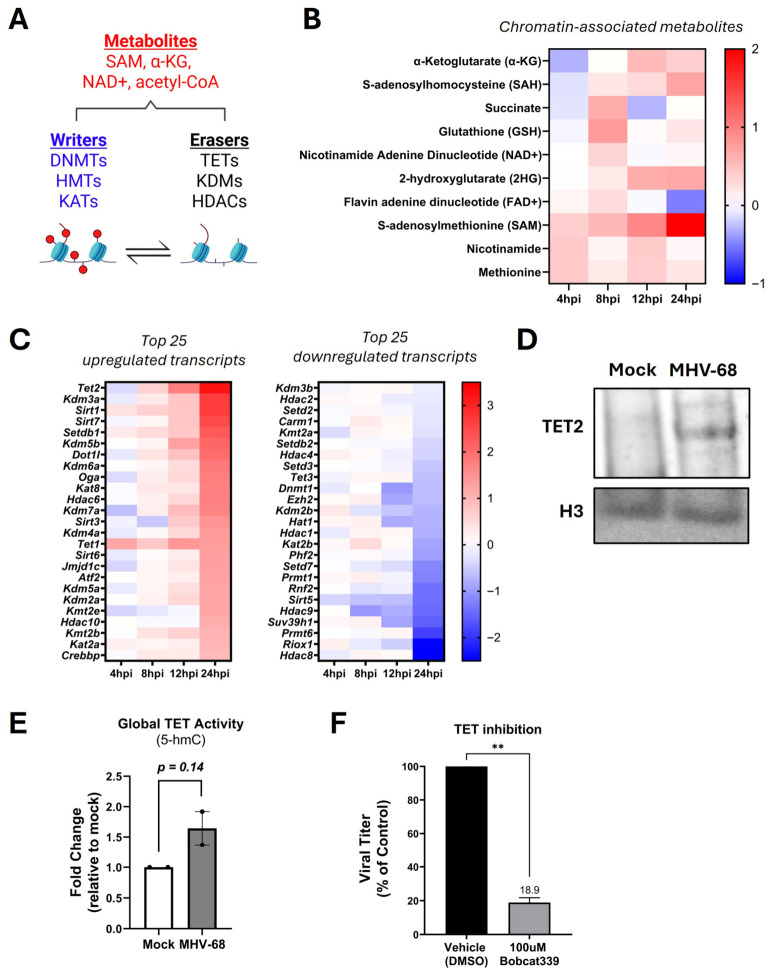
Gammaherpesvirus Lytic Infection Activates Host Chromatin Remodeling and TET-Dependent Epigenetic Pathways. (**A**) Schematic of chromatin “writers” or “erasers”. Writers: DNA methyltransferases (DNMTs) methylate cytosines, histone methyltransferases (HMTs) methylate histones, and lysine acetyltransferases (KATs) acetylate histones. Erasers: Ten-Eleven Translocation methylcytosine dioxygenases (TETs) demethylate cytosines, histone demethylases (KDMs) demethylate histones, and histone deacetylases (HDACs) deacetylate histones. The most common metabolite co-factors for chromatin writers and erasers are shown. Image created in Biorender. (**B**) Metabolomics analysis of chromatin-associated metabolite co-factors required for chromatin remodeling enzymes at 4, 8, 12, and 24 hpi in three biological replicates. The log2FC is shown, where red indicates an increased and blue indicates a decreased abundance in MHV-68-infected (MOI = 3) NIH 3T3 cells compared to mock-infected cells. (**C**) RNA-seq analysis of chromatin remodeling enzyme transcripts at 4, 8, 12, and 24 hpi in three biological replicates. The log2FC is shown, where red indicates upregulation and blue indicates downregulation in MHV-68-infected (MOI = 5) NIH 3T3 cells compared to mock-infected cells. (**D**) Western blot analysis of TET2 and H3 (control) in nuclear lysates from mock versus MHV-68-infected (MOI  =  3) NIH 3T3 cells at 24 hpi (representative image from three biological replicates). (**E**) Global TET activity measuring DNA demethylation [5-methylcytosine (5-mC) to 5-hydroxymethylcytosine (5-hmC)] by ELISA in mock- vs. MHV-68-infected (MOI = 5) NIH 3T3 cells at 24 hpi from two biological replicates. Paired Student t-test (Graphpad Prism). (**F**) NIH 3T3 cells were mock- or MHV-68-infected (MOI = 0.1) and treated with 100 µM Bobcat339 (a TET 1/2 inhibitor) or vehicle (control) for 48 hpi in three biological replicates. Viral titers were quantified by plaque assays and normalized to vehicle controls. Paired Student t-test (Graphpad Prism); **, *p* < 0.01.

At the intersection of altered metabolic and transcriptional programs are epigenetic mechanisms which utilize specific metabolites for the activity of chromatin-modifying enzymes. These metabolites act as substrates and co-factors for “writers” and “erasers”, remodeling chromatin and altering gene expression by either depositing or removing chemical modifications on DNA or histones (Figure 5A) [27,40,41]. As alterations to nucleotide biosynthesis, metabolites and chromatin regulation are tightly interconnected [40,41], we therefore sought to determine whether MHV-68 alters metabolites and host genes associated with epigenetic regulation. Our metabolomics analysis revealed coordinated changes in multiple metabolites directly linked to epigenetic regulation (Figure 5B). Among the most differentially upregulated metabolites over time was α-ketoglutarate (α-KG), a required co-substrate for Ten-Eleven Translocation (TET) and Jumonji domain-containing (JMJC) enzymes, that act as demethylases for DNA and histones, respectively (Figure 5B) [42]. Similarly, S-adenosylhomocysteine (SAH), a byproduct of S-adenosylmethionine (SAM) and a strong inhibitor of DNA and histone methylation [42], was differentially upregulated (Figure 5B).

To ascertain which host epigenetic mechanisms are transcriptionally remodeled during lytic infection, we filtered the RNA-seq dataset to only enzymes that mediate epigenetic modifications using a previously published dataset (Supplementary Table S3) [27]. Analysis of the top 25 most differentially upregulated or downregulated transcripts that peaked at 24 hpi (Figure 5C) revealed *Tet2*, which has demethylation DNA activity, and Lysine Demethylase 3A (*Kdm3a*), an enzyme crucial for removing mono- and di-methyl groups from histone 3 lysine 9 (H3K9me1/2) [43], as the most differentially expressed transcripts (Figure 5C). In contrast, various transcripts encoding epigenetic enzymes that regulate heterochromatin formation and gene silencing, such as Polycomb group proteins *Ezh2* and *Rnf2*, as well as *Suv39h1*, which methylates H3K9me3, were downregulated as MHV-68 lytic infection progressed (8–24 hpi) (Figure 5C). Moreover, we found the *Tet1* transcripts were also elevated during MHV-68 infection (Figure 5C), supporting the possibility of active DNA demethylation during lytic infection in host cells. To assess whether upregulation of TET2 also occurs at the protein level, we isolated nuclear extracts from mock- and MHV-68-infected NIH 3T3 cells (MOI = 5) at 24 hpi. Western blot analysis of nuclear lysates show upregulation of TET2 protein at 24 hpi (Figure 5D), a time point corresponding to the highest *Tet2* transcript expression.

TET family enzymes catalyze the DNA oxidation of 5-methylcytosine (5-mC) to 5-hydroxymethylcytosine (5-hmC), a stable functional DNA base modification and byproduct of TET activity [44]. To determine whether global TET activity also increased in infected cells, we harvested DNA from mock- and MHV-68-infected cells (MOI = 5) at 24 hpi and measured the levels of 5-hmC DNA by ELISA. Global 5-hmC levels increased 1.6-fold in MHV-68-infected NIH 3T3 cells relative to mock-infected controls (Figure 5E), although this did not reach statistical significance, likely due to the limitations of using a global rather than locus-specific 5-hmC assay. Despite this, the directionality of the change is consistent with increased TET expression observed at the transcript and protein levels. Overall, these data demonstrate the global alteration of metabolites and gene transcripts critical to epigenetically reprogram host cells during lytic gammaherpesvirus infection.

To examine whether TET enzymatic epigenetic activity is required for infectious viral production, NIH 3T3 cells were mock- or MHV-68-infected (MOI = 0.1) and then treated with vehicle (solvent control) or 100 µM Bobcat339, a pharmacological inhibitor of TET1 and TET2 [45,46]. Treatment with 100 µM Bobcat339 slightly reduced cell proliferation but did not induce cytotoxicity in mock-infected NIH 3T3 cells compared to vehicle-treated cells (solvent control) (Table 1). Overall, treatment of MHV-68-infected NIH 3T3 cells with 100 µM Bobcat339 resulted in a ~81% reduction in infectious virus production compared to vehicle-treated cells (*p* < 0.05) (Figure 5F). Due to slight inhibition of cell proliferation by 100 µM Bobcat339 treatment, normalization of viral titer to live cell number revealed 100 µM Bobcat339 treatment decreased infectious virus production by ~4-fold compared to vehicle-treated cells (Table 1). Taken together, these data suggest that alterations to the epigenome are required for infectious gammaherpesvirus production.

## 4. Discussion

The rewiring of host metabolism is a conserved feature during lytic gammaherpesvirus infections [11,16,47]; however, how viral-induced metabolic rewiring is coordinated with host cell transcriptional reprogramming during lytic infection remains poorly understood. In this study, we define the temporal host transcriptional landscape during lytic gammaherpesvirus infection. By integrating transcriptomic, metabolomic, and epigenetic pathways, along with pharmacological inhibition studies, we have identified a pivotal metabolic–epigenetic crosstalk that supports infectious virus production (Figure 4 and Figure 5).

Global transcriptional profiling across immediate-early (4 hpi), early (8 hpi), late (12 hpi), and very late (24 hpi) temporal stages of lytic gammaherpesvirus infection revealed widespread and progressive host gene expression changes over time (Figure 1). During early infection, there was a clear induction of pro-inflammatory cytokines and ISGs, reflecting rapid activation of innate immune pathways (Figure 1 and Figure 2). Notably, these antiviral gene signatures persisted through late stages of lytic infection, suggesting sustained immune sensing throughout lytic MHV-68 infection. These findings are consistent with previous studies demonstrating robust innate immune activation during lytic MHV-68 infection [48,49,50,51]. Importantly, analysis of the top upregulated transcripts at each time point identified additional innate genes induced during lytic MHV-68 infection that have not been previously reported, including the chemokine receptor *Cx3cr1*, type I interferon *Ifna13*, and multiple ISGs (*Irgm2*, *Oasl2*, *Mx2*, *Ifit1*, *Ifit3b*, *Mx1*, *Oas1a*, *Zbp1*, *Ifi44*, and *Ligp1*).

In addition to innate immune programs, our RNA-seq data analysis revealed the induction of host transcriptional regulators and signaling pathways that are frequently engaged during herpesvirus infection. For example, MHV-68 infection increased the transcripts of two activator protein-1 (AP-1) complex transcription factors, *Fos* and *Atf3*, a set of rapid-response transcription factors downstream of MAPK signaling that regulate gene expression (Supplementary Table S1) [52,53]. Increased *Fos* and *Atf3* expression has been linked to transcriptional control in various herpesviruses, including Human Cytomegalovirus [54,55], Kaposi’s Sarcoma Herpesvirus [56,57], Epstein–Barr Virus [58], Herpes Simplex Virus 1 [59,60,61], Herpes Simplex Virus 2 [62], Bovine Alpherpesvirus 1 [61], and Equid alphaherpesvirus 1 [61]. In addition, lytic MHV-68 infection induced the expression of key angiogenic signaling transcripts, *Vegfa* and *Kdr* (Supplementary Table S1) [63,64]. More specifically, vascular endothelial growth factor receptor 2 (*Kdr*) and vascular endothelial growth factor A (*Vegfa)* have been shown to be induced in host cells during the infection of multiple herpesviruses, including Kaposi’s Sarcoma Herpesvirus [65,66], Epstein–Barr Virus [67,68,69,70] and Human Cytomegalovirus [71].

Furthermore, our transcriptomic analysis revealed widespread remodeling of host metabolic gene expression during lytic gammaherpesvirus infection (Figure 3). These findings extend our prior metabolomics and lipidomics work which demonstrated that the rewiring of host cell lipid, glucose, and glutamine metabolism during MHV-68 infection is required for efficient viral production [11]. Importantly, this study identifies a coordinated induction of metabolic transcripts involved in central carbon metabolism, nucleotide biosynthesis, redox-associated pathways, and the PPP, revealing a previously uncharacterized layer of metabolic reprogramming during lytic MHV-68 infection.

Among the enriched metabolic programs identified in our transcriptomic analysis, the PPP emerged as a prominent upregulated metabolic pathway during lytic MHV-68 infection (Figure 4). The induction of PPP enzyme transcripts, including *G6pdx*, *Pgls*, and *Pdg*, together with increased abundance of PPP metabolic intermediates, was most pronounced during early-to-mid stages of lytic infection (8–12 hpi) (Figure 4B,C). Functional inhibition of the pentose phosphate pathway using 6-AN resulted in a ~5-fold reduction in infectious MHV-68 production (Figure 4D), indicating that the pentose phosphate pathway is required for efficient lytic gammaherpesvirus production. Consistent with this, PPP induction has been observed during productive infection by several viruses, including herpes simplex 1, adenovirus, Zika virus, and SARS-CoV-2 [72,73,74,75]. PPP induction has also been observed during latent KSHV infection and hypoxia-associated lytic EBV infection [15,76], though it has not been extensively characterized during robust gammaherpesvirus lytic replication, as we demonstrate here. In addition, previous studies have demonstrated 6-AN suppresses the replication of Hepatitis B virus, Zika virus, and Vaccinia virus [77,78,79], indicating targeting the PPP may be a broad antiviral strategy.

A paradigm shift in this study is the uncovering of an epigenetic link between metabolic rewiring and transcriptional reprogramming during productive gammaherpesvirus replication. Since the seminal discoveries linking metabolism to chromatin alterations [80] and epigenetic mechanisms to viral dormancy [81], much of the research has focused on chromatin dynamics regulating viral latency [82]. Our data expand this paradigm by demonstrating that epigenetic regulation is also active during lytic gammaherpesvirus infection. Among several metabolites known to alter chromatin-modulating enzymic activity and gene expression (Figure 5A), α-KG is often viewed as a key metabolite linking cellular metabolic state to epigenetic regulation [83]. We found α-KG is differentially upregulated upon MHV-68 infection (Figure 5B), accompanied by increased expression of *Tet1* and *Tet2* transcripts (Figure 5C), and upregulation of TET2 at the protein level (Figure 5D). While previous studies have found that TET activity regulates epigenetic control of gammaherpesvirus genomes during latency [84,85], our results suggest that global TET activity is also upregulated upon MHV-68 infection (Figure 5E), which did not reach statistical significance. Notably, TET activity was functionally required for efficient lytic viral production (Figure 5F). Pharmacological inhibition of TET1 and TET2 activity with Bobcat339 significantly reduced infectious MHV-68 production (Figure 5F), indicating that TET-dependent DNA demethylation is necessary to support productive gammaherpesvirus replication. These results reveal the duality of TET function during latent and lytic phases of viral infection and point to the importance of studying chromatin remodeling complexes during lytic gammaherpesvirus infection.

While this study integrates transcriptomic, metabolomic, and pharmacological analyses to define host pathways supporting lytic MHV-68 infection, several limitations should be considered. First, although we observed widespread transcriptional remodeling during infection, changes in transcript abundance do not necessarily correspond with protein expression changes or enzymatic activity. Further studies incorporating proteomic profiling and metabolic flux analysis will further refine the functional contribution of the pathways identified here. Second, all experiments were conducted in NIH 3T3 fibroblasts under controlled in vitro infection conditions. Although NIH 3T3 cells enable robust lytic replication, metabolic and epigenetic responses may differ in vitro in other cell types or in in vivo settings where immune and tissue-specific microenvironments influence infection dynamics. Third, although the global DNA 5-hmC levels showed a modest yet not statistically significant increase in MHV-68-infected cells, the consistent upregulation of *Tet1* and *Tet2* transcripts (Figure 5C) and TET2 protein (Figure 5D), along with the requirement of TET activity for infectious MHV-68 production (Figure 5F), supports a functional role for TET activity during lytic replication. Together, these data identify TET-mediated DNA demethylation as a key candidate regulator of gammaherpesvirus infection and future studies will be required to define specific epigenetic targets.

## 5. Conclusions

Overall, we define a metabolic–epigenetic crosstalk that supports productive gammaherpesvirus replication. The temporal RNA-seq dataset generated here provides a resource for the identification of additional host pathways altered during lytic infection that may be explored for future therapeutic intervention. Importantly, MHV-68 serves as a well-established experimental system that provides a lytic gammaherpesvirus surrogate model to identify host metabolic and epigenetic pathways that may also be modulated during human lytic replication. Inhibition of either the PPP or TET enzymatic activity significantly reduced lytic gammaherpesvirus production, demonstrating that these host pathways are required for infectious virus production. Whether metabolic changes initiate chromatin alterations, or epigenetic reprogramming drives downstream metabolic adaptation represents an important question for future investigation. Currently, there are no approved antiviral treatments for human gammaherpesviruses. Because the PPP and TET enzymes are host-encoded rather than virally encoded, targeting these pathways may offer broad antiviral strategies against human gammaherpesviruses while reducing the likelihood of viral resistance. Taken together, these data establish viral dependence on host metabolic–epigenetic reprogramming as a critical determinant of efficient lytic gammaherpesvirus production and its potential as a therapeutic target against human gammaherpesvirus infections.

## Figures and Tables

**Table 1:** Normalized fold-change reduction in virus production in treated compared to control-treated MHV-68-infected NIH3T3 cells.

Condition	Total Cells (Average)	Live Cells (Average)	Viability (%)	pfu/mL (Average)	pfu/mL Live Cell Number RATIO	Normalized Reduction in Virus Production (Fold Change)
6-Aminonicotinamide (6-AN)						
Mock (DMSO)	2,386,000	2,357,000	99%			
Mock (35 µM 6-AN)	1,021,000	991,000	97%			
MHV-68 (DMSO)	1,230,000	1,193,000	97%	14,025	1.18 × 10^−2^	
MHV-68 (35 µM 6-AN)	976,000	920,000	94%	2142	2.33 × 10^−3^	5.1
Bobcat339						
Mock (DMSO)	1,690,000	1,690,000	100%			
Mock (100 µM Bobcat339)	1,137,000	1,133,000	100%			
MHV-68 (DMSO)	1,035,000	1,022,000	99%	57,917	5.67 × 10^−2^	
MHV-68 (100 µM Bobcat339)	693,000	688,000	99%	10,333	1.50 × 10^−2^	3.8

## Data Availability

The RNA-seq data has been deposited in the NCBI Gene Expression Omnibus (GEO) under the accession number GSE314414.

## Supplementary Materials

The following supporting information can be downloaded at: https://www.mdpi.com/article/10.3390/v18050574/s1, Table S1: RNA-seq analysis of NIH 3T3 cells during lytic MHV-68 infection; Table S2: RNA-seq analysis of Metabolic Genes (KEGG 01100 module) during lytic MHV-68 infection of NIH 3T3 cells; Table S3: RNA-seq analysis of chromatin-modulating enzymes during lytic MHV-68 infection of NIH 3T3 cells; Figure S1: Host Transcriptional Pathways Downregulated During Gammaherpesvirus Lytic Infection; Figure S2: Metabolic Pathways Downregulated During Lytic Gammaherpesvirus.

## Abbreviations

The following abbreviations are used in this manuscript:

5-mC	5-methylcytosine
5-hmC	5-hydroxymethylcytosine
6-AN	6-aminonicotinamide
α-KG	α-ketoglutarate
DEG	Differential gene expression
DMEM	Dulbecco’s Modified Eagle Medium
DMSO	Dimethyl Sulfoxide
EBV	Epstein–Barr Virus
ELISA	Enzyme-Linked Immunosorbent Assay
GEO	Gene Expression Omnibus
HPI	Hours Post-Infection
KSHV	Kaposi’s Sarcoma-associated Herpesvirus
log2FC	log 2 fold-change
MHV-68	Murine Herpesvirus 68
MOI	Multiplicity of Infection
padj	*p*-value adjusted
PCA	Principal component analysis
PFUs	Plaque-forming units
PPP	Pentose Phosphate Pathway
RNA-seq	RNA-sequencing
SAH	S-adenosylhomocysteine
SAM	S-adenosylmethionine
TET	Ten-Eleven Translocation
