# Supplementary material for: Targeting Host Metabolic and Epigenetic Rewiring Blocks Lytic Gammaherpesvirus Production
Source: Viruses. 2026 May 19;18(5):574. doi: 10.3390/v18050574 (PMC13211458; doi:10.3390/v18050574)
Supplement: Supplementary file 1 [file viruses-18-00574-s001.zip › Supplementary Figures.pdf]

Supplementary Figure S1

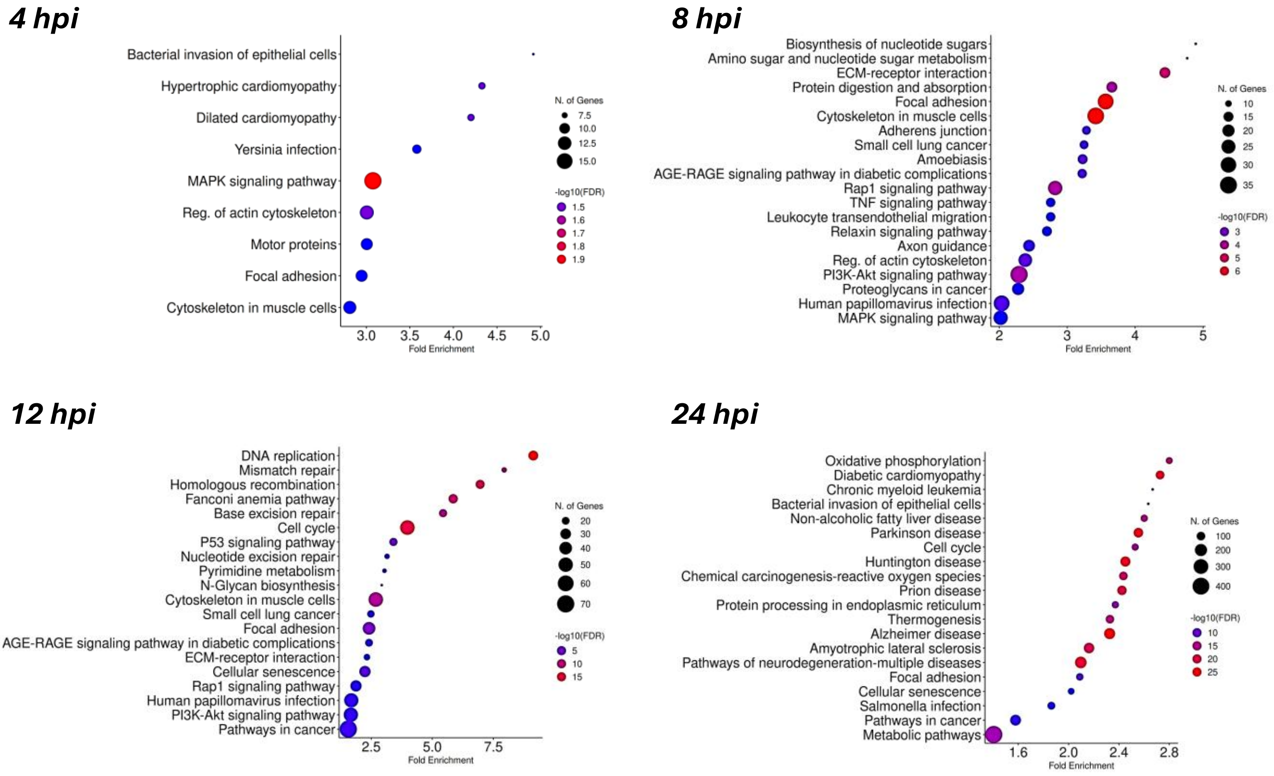

Figure S1: Host Transcriptional Pathways Downregulated During Gammaherpesvirus Lytic Infection. Gene ontology KEGG pathway enrichment analysis of downregulated DEGs in MHV-68-infected NIH 3T3 cells vs mock-infected cells (log2FC $\leq$ -0.5; padj < 0.05) at A) 4 hpi, B) 8 hpi, C) 12 hpi, and D) 24 hpi using ShinyGO version 0.85. Dot size represents the number of mapped genes and dot color indicates −log10(FDR).

Supplementary Figure S2

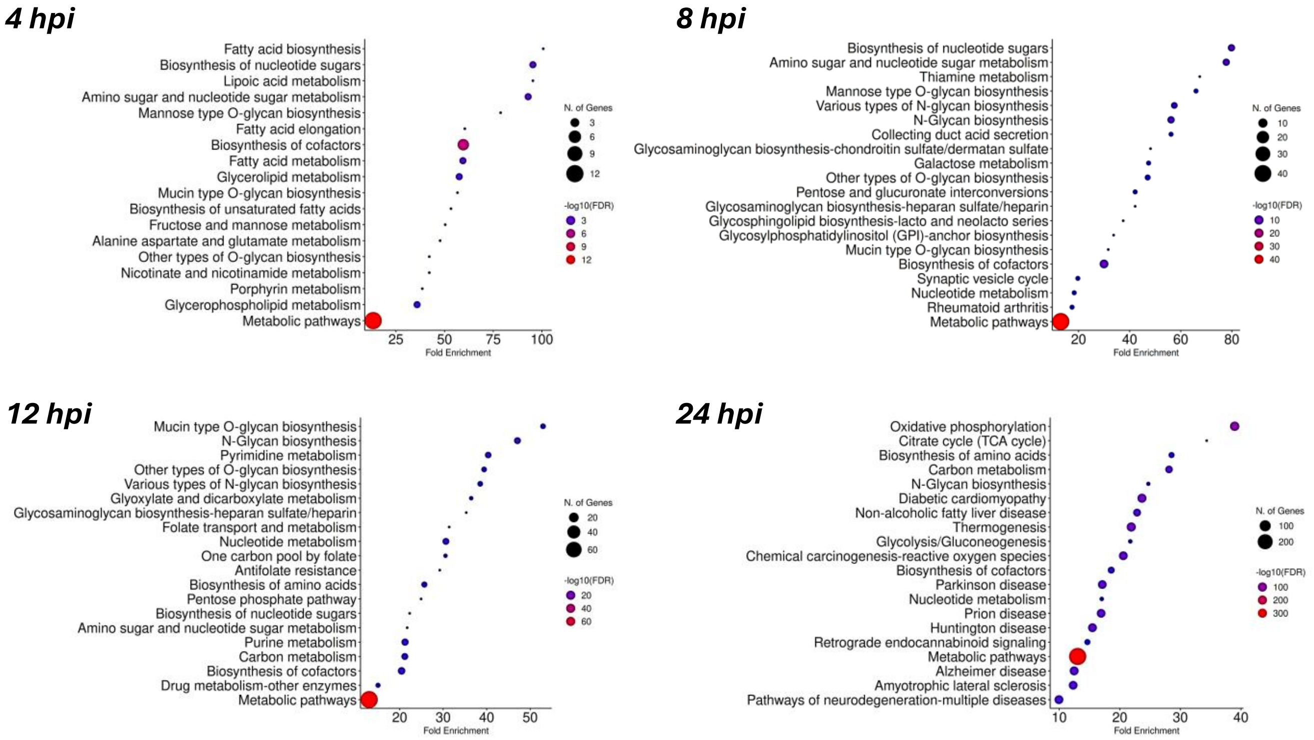

Figure S2: Metabolic Pathways Downregulated During Lytic Gammaherpesvirus Infection. Gene ontology KEGG pathway enrichment analysis of downregulated metabolic DEGs (log2FC $\leq$ +0.5; padj < 0.05) at E) 4 hpi, F) 8 hpi, G) 12 hpi, and H) 24 hpi using ShinyGO version 0.85. Dot size represents the number of mapped genes and dot color indicates −log10(FDR).
